# Prepapillary Vascular Loops Complicated by Suspected Macroaneurysm Rupture

**DOI:** 10.1155/2014/157242

**Published:** 2014-09-23

**Authors:** Kei Akaiwa, Yoshinori Mitamura, Takashi Katome, Kentaro Semba, Mariko Egawa, Takeshi Naito

**Affiliations:** Department of Ophthalmology, Institute of Health Biosciences, The University of Tokushima Graduate School, 3-18-15 Kuramoto-cho, Tokushima 770-8503, Japan

## Abstract

We present a case of prepapillary vascular loops complicated by a suspected macroaneurysm rupture which was treated with intravitreal bevacizumab (IVB). A 62-year-old woman presented with decreased vision and myodesopsia in her left eye. Her best-corrected visual acuity (BCVA) was 0.6 in the left eye. Fundus examination disclosed an elevated, round, and reddish lesion, retinal hemorrhage at the superior aspect of the optic disc, retinal opacification along the superior branch retinal artery, and a small vitreous hemorrhage. Optical coherence tomography showed a serous retinal detachment, and indocyanine green angiography demonstrated prepapillary vascular loops and a hypofluorescent area with hyperfluorescent margins. These findings suggested the presence of a macroaneurysm. No filling of the dye in the aneurysm-like dilatation suggested a blockage of the lumen with a thrombus which might be associated with a branch retinal artery occlusion (BRAO). A diagnosis of prepapillary vascular loops complicated by a suspected macroaneurysm rupture and BRAO was made. Because of a persistent serous retinal detachment, IVB was performed. One month later, the BCVA improved to 1.0. Fundus examination disclosed an organized yellowish-white macroaneurysm and resolution of the serous retinal detachment. We recommend careful monitoring of patients with prepapillary vascular loops because of complications such as macroaneurysm rupture and BRAO.

## 1. Introduction

Prepapillary vascular loop is a variant of the normal retinal vasculature seen on and around the optic disc [1–4]. Their prevalence is estimated at 1 : 2000 to 1 : 9000 eyes, but this is likely an underestimation because many patients with prepapillary vascular loop are asymptomatic [5, 6]. They are usually detected incidentally on routine fundus examinations [7] and are usually unilateral [5]. Prepapillary vascular loops have been reported to be associated with branch retinal artery occlusion (BRAO), amaurosis fugax, recurrent vitreous hemorrhage, subretinal hemorrhage, and hyphema [2, 7–12].

To the best of our knowledge, there is only one report of an eye with prepapillary vascular loops complicated by a macroaneurysm [13]. We report a case of prepapillary vascular loops complicated by a suspected macroaneurysm rupture and serous retinal detachment which were treated with intravitreal bevacizumab (IVB).

## 2. Case Presentation

A 62-year-old woman presented with complaints of decrease in vision and myodesopsia in her left eye of 1-month duration. There was no relevant ocular or medical history. She had no history of hypertension, hyperlipidemia, and diabetes mellitus. Her best-corrected visual acuity (BCVA) was 1.5 in the right eye and 0.6 in the left eye. The intraocular pressure was 10 mmHg in both eyes. Slit-lamp examination of both eyes and fundus examination of the right eye were unremarkable.

**Figure 1:**
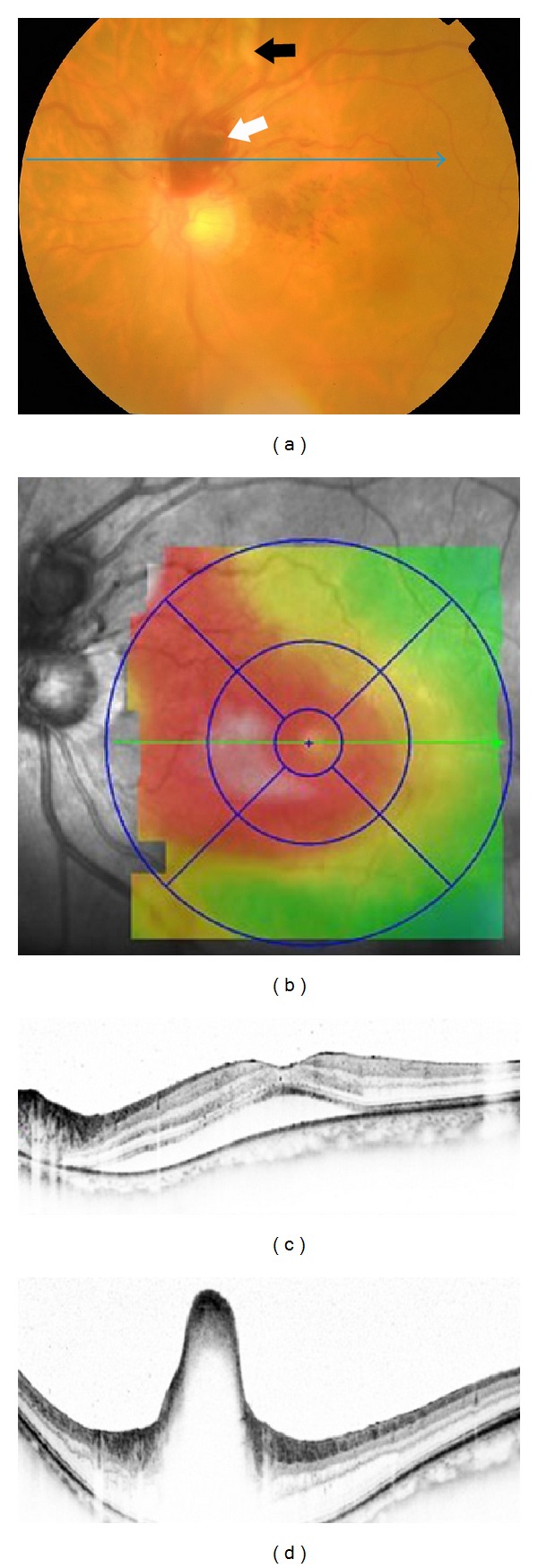
Fundus photograph and spectral-domain optical coherence tomographic (SD-OCT) images of the left eye at the initial visit. (a) Fundus photograph showing an elevated, round, and reddish lesion at the superior aspect of the optic disc (white arrow). Retinal opacification is evident along the course of the superior branch retinal artery (black arrow). Blue arrow indicates the direction of the SD-OCT scan of (d). A mild vitreous opacity is visible. Nerve fiber layer hemorrhages are present superonasal to fovea. (b) Retinal thickness map of SD-OCT shows the retinal thickening extends from the parapaillary region to the macula. (c) SD-OCT scan through the fovea shows serous retinal detachment involving fovea. (d) SD-OCT scan through the red lesion shows marked elevation of the retinal surface.

**Figure 2:**
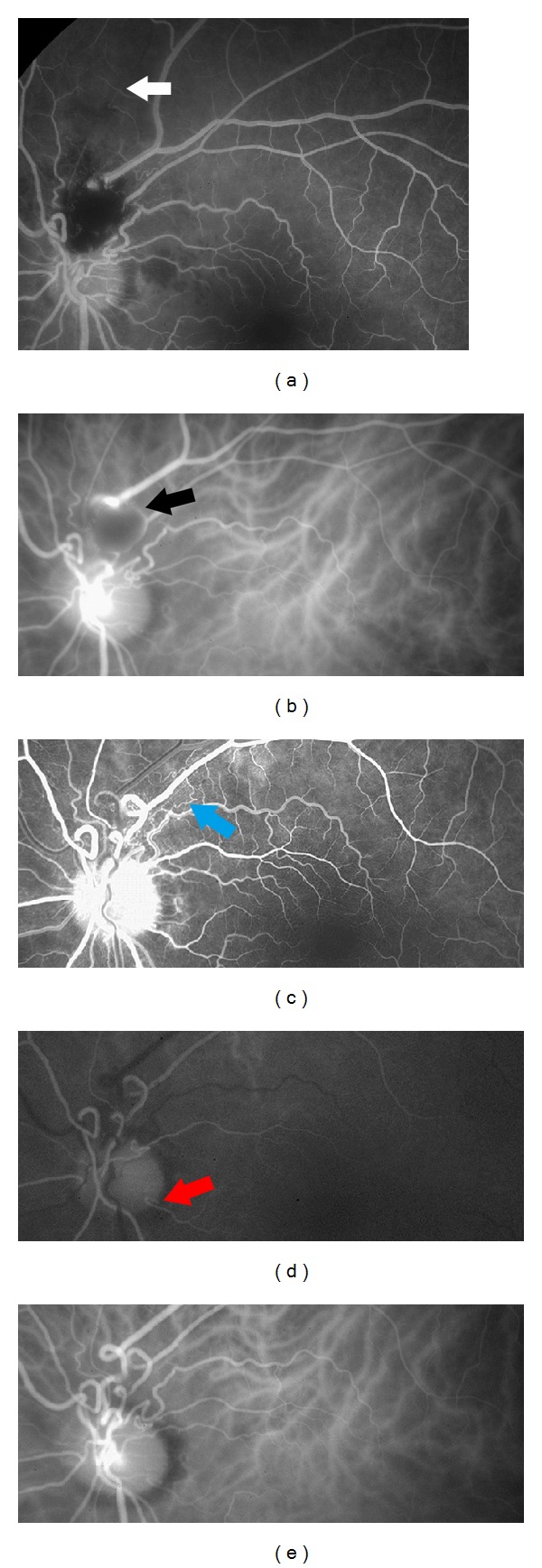
Fluorescein angiographic (FA) and indocyanine green angiographic (ICGA) images at the initial visit ((a), (b)) and one year after the initial visit ((c), (d), and (e)). (a) FA at the initial visit shows incomplete perfusion of the superior branch retinal artery (white arrow) indicating a branch retinal artery occlusion. Vascular loops can be seen. (b) ICGA at the initial visit demonstrates prepapillary vascular loops and a round hypofluorescent area with hyperfluorescent margin (black arrow) suggesting the presence of a macroaneurysm. No filling of the dye in the aneurysm-like dilatation suggests a blockage of the lumen with a thrombus. However, communication with this aneurysm-like dilatation and the retinal artery is unclear. (c) Early venous phase FA one year after the initial visit shows venous and arterial prepapillary loops with spiral turns. Laminar flow is obvious within the venous loops. Fine collateral vessels (dilatation of retinal capillary vessels; blue arrow) are seen at the superotemporal aspect of the optic disc. (d) Early phase ICGA one year after the initial visit shows cilioretinal artery (red arrow). (e) Late phase ICGA clearly shows vascular loop formation and no aneurysm-like lesion at the superior aspect of the optic disc.

Fundus examination of the left eye disclosed an elevated, round, and reddish lesion and a retinal hemorrhage at the superior aspect of the optic disc. In addition, there were retinal opacification along the superior branch retinal artery and a small vitreous hemorrhage (Figure 1(a)). Spectral-domain optical coherence tomography (SD-OCT) showed a serous retinal detachment involving fovea and marked elevation of the retinal surface in the reddish lesion (Figures 1(c) and 1(d)). The SD-OCT map of the retinal thickness showed a thickening extending from the parapapillary region to the macula (Figure 1(b)). Fluorescein angiography (FA) showed incomplete perfusion of the superior branch retinal artery indicating the presence of a BRAO (Figure 2(a)). Because visual field tests were not performed, a presence of visual field defect corresponding BRAO was uncertain. The vascular loops could be seen on FA but did not show any dye leakage even in the late phase of FA. Indocyanine green angiography (ICGA) demonstrated the prepapillary vascular loops and a round hypofluorescent area with hyperfluorescent margins (Figure 2(b)). These findings suggested the presence of a macroaneurysm. The lack of filling of the aneurysm-like round dilatation suggested a blockage of the lumen with a thrombus which was considered to be associated with the BRAO. However, communication with the aneurysm-like dilatation and the retinal artery was unclear.

**Figure 3:**
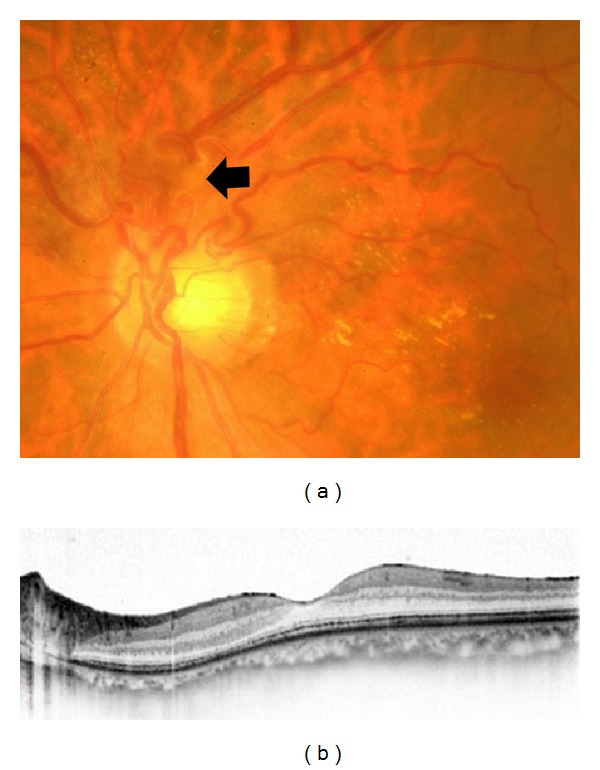
Fundus photograph and spectral-domain optical coherence tomographic (SD-OCT) image of the left eye at one month after intravitreal bevacizumab. (a) Fundus photograph indicates venous and arterial prepapillary loops with spiral turns and a slightly organized, yellowish-white lesion at the superior aspect of the optic disc (black arrow). Hard exudates are observed around the macula. (b) SD-OCT scan through the fovea shows no serous retinal detachment.

A diagnosis of prepapillary vascular loops complicated by a suspected macroaneurysm rupture and BRAO was made. One month after the initial presentation, IVB was performed because the serous retinal detachment persisted. This was an off-label use of bevacizumab, and the procedures were approved by the Institutional Review Board of Tokushima University Hospital. The procedures used for the treatments conformed to the tenets of the Declaration of Helsinki, and an informed consent was obtained from the patient after the benefits, potential risks, and alternative treatments were discussed. One month after IVB, the BCVA improved to 1.0. Fundus examination disclosed venous and arterial prepapillary loops with spiral turns, a slightly organized, yellowish-white lesion at the superior aspect of the optic disc, and hard exudates around the macula (Figure 3(a)). SD-OCT showed a resolution of serous retinal detachment (Figure 3(b)).

One year after the initial presentation, the patient maintained a BCVA of 1.0 in the left eye, and fundus examination disclosed a completely organized, white macroaneurysm. FA clearly showed venous and arterial prepapillary loops with spiral turns (Figure 2(c)). Fine collateral vessels (dilatation of retinal capillary vessels) were seen at the superotemporal aspect of the optic disc. ICGA showed a cilioretinal artery (Figure 2(d)) and no aneurysm-like lesion at the superior aspect of the optic disc (Figure 2(e)).

## 3. Discussion

Kubo et al. [13] reported a case of bilateral optic disc macroaneurysm associated with acquired prepapillary vascular loops. Their patient had vitreous hemorrhages in both eyes and a BRAO in the left eye. Our case had prepapillary vascular loops possibly complicated by a ruptured macroaneurysm. ICGA showed no filling of the aneurysmal dilatation suggesting that autothrombosis had occurred. This autothrombosis may have been the main cause of the BRAO [14].

The macroaneurysm in our case was most likely arterial. A communication between the aneurysm-like dilatation and the retinal artery was not clear in the ICGA images. However, a combination of a fresh BRAO suggested that it was probably an arteriolar macroaneurysm.

The majority of prepapillary vascular loops are thought to be congenital in origin [2]. Acquired prepapillary arterial loops have been described following central retinal artery occlusion [15] and in a patient whose initial finding was multiple cotton wool spots of unknown origin [16]. Whether the prepapillary vascular loops in our case were congenital or acquired could not be definitively determined. However, previous cases with congenital prepapillary vascular loops usually had only one branch artery protruding into the vitreous cavity [5]. This would suggest that the multiloops in the present case might not be congenital. Bronner et al. [17] described several features to differentiate the congenital and acquired prepapillary vascular loops. Most of acquired prepapillary loops develop secondary to central or branch retinal venous obstruction or optic nerve tumor [17]. The present case reported no relevant ocular history. However, fine collateral vessels at the superotemporal aspect of the optic disc suggested a presence of an old branch retinal venous obstruction, although there were no findings of retinal venous dilation, tortuosity, or peripheral retinal hemorrhages.

Approximately 95% of prepapillary loops are arterial based on FA findings, and loops of venous origin are extremely rare [2, 3]. Matsui et al. [18] performed FA on 7 patients with prepapillary loops and found that all the loops were arterial. Degenhart et al. [2] found only one venous loop in a series of 21 eyes with prepapillary vascular loops. Although the exact incidence of venous loops has not been determined, these reports suggest that the venous loops occur much less commonly than arterial loops. Several venous and arterial prepapillary loops as seen in our patient have been reported only by Teramoto et al. [3] and seem to be much rarer.

Prepapillary vascular loops frequently accompany unusual vascular pattern such as cilioretinal artery as was present in our case [2, 3, 12, 19]. Teramoto et al. [3] reported bilateral prepapillary venous and arterial loops accompanied by a cilioretinal artery, intertwined retinal artery and vein, and trifurcation of a retinal vein. A cilioretinal artery was most frequently reported in association with vascular loops [2, 11, 12] and has been described in up to 75% of eyes with prepapillary loops [2]. Soltau et al. [20] reported a case of prepapillary arterial loop accompanied by venous retinal macrovessels which were congenital vessels crossing the horizontal raphe in the macular area.

Whether a congenital prepapillary vascular loop is derived from the hyaloid or retinal system has been debated [5]. Earlier hypotheses suspected that the prepapillary vascular loops were remnants of the hyaloid artery. However, an anatomic study [21] and association with BRAO strongly supported the origin of prepapillary vascular loops from the retinal arterial system. Shakin et al. [21] reported an anatomic study of prepapillary vascular loops. The loops communicated with the retinal arterial system and did not have an internal elastic lamina as is the case in normal retinal artery. Additionally, the perivascular connective tissue matrix of the vascular loops contained less hyaluronic acid than the vitreous one [21]. These findings seem to support the embryologic derivation of congenital prepapillary vascular loops from the retinal arterial system rather than from the hyaloid artery [4].

In conclusion, careful observation and monitoring of patients with prepapillary vascular loops is necessary because of the possibility of complications such as macroaneurysm rupture, BRAO, and vitreous hemorrhage. Prepapillary vascular loops should be considered in the differential diagnosis when retinal hemorrhage extends from the parapapillary region.
